# Unravelıng non-canonıcal Wnt sıgnalıng ın neural development and dısorders: a revıew of Wnt/PCP and Wnt/ca^²⁺^ pathways

**DOI:** 10.1007/s11033-026-12120-2

**Published:** 2026-06-10

**Authors:** Ahmet Alperen Palabiyik, Esra Palabiyik

**Affiliations:** 1 Faculty of Health Sciences, Department of Nursing, Ardahan University, Ardahan, Türkiye; 2 Department of Molecular Biology and Genetics, Ağrı İbrahim Çeçen University, Ağrı, Türkiye

**Keywords:** Wnt/PCP, Wnt/Ca²⁺, Non-canonical Wnt signaling, Planar cell polarity, Frizzled receptors

## Abstract

Non-canonical Wnt signaling pathways, particularly the Planar Cell Polarity (Wnt/PCP) and Wnt/Ca²⁺ branches, play critical roles in regulating neural development and maintaining cellular homeostasis within the nervous system. This review provides a comprehensive evaluation of the molecular and cellular functions of these pathways, with a focus on their involvement in cytoskeletal dynamics, cellular polarity, and calcium signaling. Non-canonical Wnt signaling is essential for key neurodevelopmental processes, including neural tube closure, neuronal migration, axon guidance, and synaptic plasticity, while its dysregulation contributes to the pathogenesis of both neurodevelopmental and neurodegenerative disorders such as autism spectrum disorder, spina bifida, Alzheimer’s disease, and Parkinson’s disease. Particular emphasis is placed on the role of Dishevelled (DVL) as a shared intracellular signaling mediator across canonical and non-canonical Wnt pathways, as well as on the context-dependent activation of downstream effectors including Rho GTPases, protein kinase C (PKC), calcium/calmodulin-dependent kinase II (CaMKII), and nuclear factor of activated T-cells (NFAT). In addition, emerging experimental platforms, including CRISPR/Cas9 gene editing and human brain organoids, are highlighted for their utility in dissecting pathway-specific mechanisms and advancing translational applications. Overall, this review underscores the importance of non-canonical Wnt signaling as a dynamic and context-dependent regulatory network and discusses its potential as a target for precision-based therapeutic strategies in neurological disorders.

## Introduction

Wingless/Integrated (Wnt) signaling pathways represent an evolutionarily conserved family of molecular mechanisms that govern crucial aspects of embryonic development, tissue homeostasis, and cell fate determination [1]. Traditionally, Wnt signaling has been divided into two major branches: the canonical (β-catenin-dependent) and non-canonical (β-catenin-independent) pathways [2]. While the canonical Wnt pathway has been extensively studied due to its role in transcriptional regulation and oncogenesis, non-canonical Wnt signaling remains comparatively underexplored despite its pivotal role in regulating cellular dynamics, polarity, and morphogenetic movements particularly in the nervous system [3].

Among non-canonical branches, two prominent pathways have emerged: the Planar Cell Polarity (PCP) pathway and the Wnt/Ca²⁺ pathway [4]. The PCP pathway orchestrates the spatial orientation of cells within the plane of a tissue, a process fundamental to coordinated neural tube closure, neuronal migration, and axonal pathfinding [5]. The The Wnt/Ca²⁺ pathway modulates intracellular calcium fluxes. It also regulates key downstream effectors, including protein kinase C (PKC), calcium/calmodulin-dependent kinase II (CaMKII), and nuclear factor of activated T-cells (NFAT). Through these mechanisms, it influences synaptic plasticity, gliogenesis, and neuroinflammation [6].

Recent studies have revealed that disruptions in these non-canonical pathways are implicated in a spectrum of neurological disorders, ranging from neurodevelopmental conditions like autism spectrum disorder and spina bifida, to neurodegenerative diseases such as Alzheimer’s and Parkinson’s disease [7]. However, the underlying molecular mechanisms remain incompletely understood, and a comprehensive synthesis of current knowledge is lacking in the field [8].

In parallel with these mechanistic insights, accumulating clinical and translational evidence has further strengthened the link between non-canonical Wnt signaling and neurological disorders. Recent studies have demonstrated that dysregulation of Wnt/PCP signaling contributes to impaired neuronal connectivity and neurodevelopmental abnormalities, while aberrant Wnt/Ca²⁺ signaling is associated with calcium-dependent neurotoxicity, synaptic dysfunction, and neuroinflammatory processes observed in neurodegenerative diseases [9, 10]. These findings highlight the growing clinical relevance of non-canonical Wnt pathways and underscore their involvement in disease-specific molecular networks within the central nervous system.

This review aims to unravel the roles of Wnt/Planar Cell Polarity (Wnt/PCP) and Wnt/Ca²⁺ signaling pathways in neural development and disease, with an emphasis on their molecular architecture, functional roles in neurogenesis and synaptic formation, and their emerging links to pathological conditions. By providing a comparative overview of non-canonical versus canonical Wnt signaling in neural contexts, we hope to shed light on unresolved questions and stimulate future research directions in developmental neurobiology and neurotherapeutics.

## Database search and methodology

This narrative review was conducted through a structured and comprehensive search of the scientific literature to investigate the roles, mechanisms, and clinical implications of non-canonical Wnt signaling specifically the Wnt/PCP and Wnt/Ca²⁺ pathways in neural development and disease. The review also aimed to explore therapeutic opportunities and experimental platforms targeting these pathways. A systematic literature search was performed across major scientific databases, including PubMed, Web of Science and Scopus (Elsevier). The search covered publications between January 2011 and April 2025. It included peer-reviewed research articles, high-impact narrative and systematic reviews, preclinical in vivo and in vitro studies, and relevant translational reports. Only articles published in English in peer-reviewed journals were considered. The following search terms were used in combination and with Boolean operators where appropriate: “non-canonical Wnt signaling AND neurodevelopment”, “FZD neuron” AND “noncanonical neuron”, “Wnt/PCP pathway AND neural migration”, “Wnt/Ca²⁺ pathway AND synaptic plasticity”, “Dishevelled AND neuron polarity”, “VANGL1/2 AND cortical layering”, “ROR2 AND neurogenesis”, “Wnt5a OR Wnt11 AND neurological disorders”, “non-canonical Wnt AND Alzheimer OR autism OR schizophrenia”, “Wnt/Ca²⁺ signaling AND therapeutic targeting”. “FZD neuron” and “noncanonical neuron”.

To further clarify how these terms were systematically combined, explicit Boolean search strategies were applied as follows: To enhance transparency and reproducibility, explicit Boolean search strategies were constructed by grouping related keywords into conceptual blocks. These blocks were combined using Boolean operators (AND, OR) as follows:

Block 1 (Pathway focus):

non-canonical Wnt signaling” OR “Wnt/PCP pathway” OR “Wnt/Ca²⁺ pathway.

Block 2 (Neurobiological processes):

neurodevelopment” OR “neuronal migration” OR “axon guidance” OR “synaptic plasticity.

Block 3 (Disease context):

neurological disorders” OR “Alzheimer’s disease” OR “Parkinson’s disease” OR “autism spectrum disorder” OR “schizophrenia.

Block 4 (Molecular components):

Dishevelled” OR “Frizzled receptors” OR “ROR2” OR “VANGL.

These blocks were combined using the following Boolean structures:

(Block 1) AND (Block 2).

(Block 1) AND (Block 3).

(Block 4) AND (Block 2).

Additional targeted searches were performed using:

(“Wnt/Ca²⁺ signaling”) AND (“calcium homeostasis” OR “NFAT” OR “CaMKII” OR “PKC”).

AND (“synaptic function” OR “neuroinflammation”).

Search strategies were adapted for each database (PubMed, Web of Science, and Scopus) based.

on database-specific indexing systems and syntax requirements.

*Inclusion criteria*:

- Studies investigating Wnt/PCP or Wnt/Ca²⁺ signaling in the context of neurodevelopment, neuronal migration, or synaptic function.
- Articles exploring the role of non-canonical Wnt effectors (e.g., DVL, PKC, CaMKII, NFAT) in health and disease.
- Research utilizing animal models (e.g., zebrafish, mice) or human cell-based systems (e.g., neural progenitor cells, induced pluripotent stem cells (iPSCs) derived organoids).
- Publications evaluating therapeutic interventions or signaling modulators (e.g., DVL inhibitors, calcium pathway regulators).

*Exclusion criteria*:

- Non-peer-reviewed sources, including preprints, conference abstracts, or editorial commentaries.
- Articles not directly addressing non-canonical Wnt signaling or lacking neurobiological relevance.
- Studies with insufficient mechanistic depth or lacking experimental validation.

Additionally, manual reference screening was performed on key review papers and primary studies to identify relevant literature not captured during the initial database queries.

The selection process emphasized methodological rigor, mechanistic insight, and translational relevance. This approach ensured a coherent and comprehensive synthesis of the current state of knowledge on non-canonical Wnt signaling in neural contexts and identified emerging areas of research and therapeutic potential.

## Population: the developing and diseased nervous system

The nervous system is a complex and highly organized structure whose development depends on precise regulation of cellular polarity, migration, and morphogenetic patterning [11]. During embryogenesis, neural progenitor cells undergo tightly coordinated spatial and temporal changes that give rise to functional neural circuits [12]. Disruptions in these processes often underlie developmental anomalies and neuropsychiatric conditions [13]. Emerging evidence suggests that non-canonical Wnt pathways specifically Wnt/PCP and Wnt/Ca²⁺ are critical regulators of these neurodevelopmental events, exerting influence at cellular, tissue, and system levels [14].

### Neural tissue vulnerability to polarity disruption

PCP is essential for organizing neuroepithelial cells within the plane of a tissue, a prerequisite for neural tube closure and the establishment of body axes [5, 11]. Similarly, calcium signaling modulated by Wnt/Ca²⁺ is involved in coordinating intracellular signaling cascades required for synaptic communication and glial cell differentiation [4]. The developing brain is particularly sensitive to perturbations in these pathways, as even minor disruptions in PCP components such as Van Gogh-like (VANGL) or Dishevelled (DVL) can lead to profound structural defects [15].

In the mature nervous system, non-canonical Wnt signals continue to modulate cellular polarity, axonal alignment, and circuit reorganization, particularly during injury, neurogenesis, or neuroinflammation [16]. This ongoing regulatory role suggests that the “population” influenced by these pathways is not limited to embryonic development but also extends to plasticity and repair processes in adulthood [17].

### Biological systems and disease contexts associated with non-canonical Wnt dysfunction

Deficiencies in Wnt/PCP signaling have been directly associated with neural tube defects (NTDs), such as spina bifida and anencephaly, due to failure of convergent extension movements [18]. Moreover, Wnt/PCP disruption has been linked to schizophrenia, autism spectrum disorder (ASD), and intellectual disability, possibly via altered neuronal migration and connectivity [19].

On the other hand, Wnt/Ca²⁺ dysregulation is implicated in Alzheimer’s disease, particularly through calcium-mediated excitotoxicity and NFAT pathway hyperactivation [20]. This suggests that the target population includes not only individuals undergoing neurodevelopment but also those suffering from neurodegenerative diseases [21].

In summary, the population influenced by Wnt/PCP and Wnt/Ca²⁺ signaling encompasses both the developing nervous system, where these pathways guide cellular architecture and connectivity, and the diseased nervous system, where their dysfunction may initiate or exacerbate pathological processes.

## Intervention: mechanistic insights into Wnt/PCP and Wnt/Ca²⁺ pathways

Non-canonical Wnt signaling diverges from the canonical β-catenin-dependent pathway by activating intracellular cascades that primarily regulate cytoskeletal organization, cell polarity, and calcium dynamics [1, 22]. The two major branches Wnt/PCP and Wnt/Ca²⁺ pathways serve as critical modulators of neurodevelopment, influencing morphogenetic processes at both the cellular and tissue levels [23]. These pathways are initiated by specific Wnt ligands (e.g., Wnt5a, Wnt11) and Frizzled receptors, but transduce signals through alternative downstream effectors such as Rho Guanosine Triphosphatases (GTPases), c-Jun N-terminal Kinase (JNK), PKC, CaMKII, and NFAT [24, 25].

### Wnt/PCP pathway: molecular architecture and functional overview

The Wnt/PCP pathway regulates the orientation and alignment of cells within the plane of a tissue, a process essential for key neurodevelopmental events such as neural tube closure, neuronal migration, and axon guidance [26]. This pathway is activated by non-canonical Wnt ligands including Wnt5a and Wnt11 which bind to Frizzled (Fz) receptors in coordination with co-receptors such as Receptor Tyrosine Kinase-Like Orphan Receptor 2 (ROR2) or Protein Tyrosine Kinase 7 (PTK7) [27]. Ligand–receptor engagement initiates a cascade of intracellular signaling events centered around the scaffold protein DVL, a key intracellular mediator shared by both canonical and non-canonical Wnt signaling pathways [28]. In the context of Wnt/PCP signaling, DVL recruits a set of core PCP components that coordinate cytoskeletal reorganization and cell polarity [29]. Recent studies further demonstrate that non-canonical Wnt/PCP signaling dynamically regulates actin remodeling and cell polarity during neural development through context-dependent activation of small Rho GTPases and associated downstream effectors [9, 30–32].

Among these core proteins, VANGL1/2 are responsible for polarized membrane localization, while Prickle (PK) contributes to asymmetric protein distribution along the apical basal axis [33]. Daam1 serves as a linker between DVL and small Rho GTPases, particularly RhoA and Rac1, facilitating cytoskeletal dynamics and morphogenetic rearrangements [34]. The downstream effectors RhoA, Rac1, and JNK orchestrate actin remodeling and support transcription-independent regulation of cell behavior [35].

**Fig. 1:**
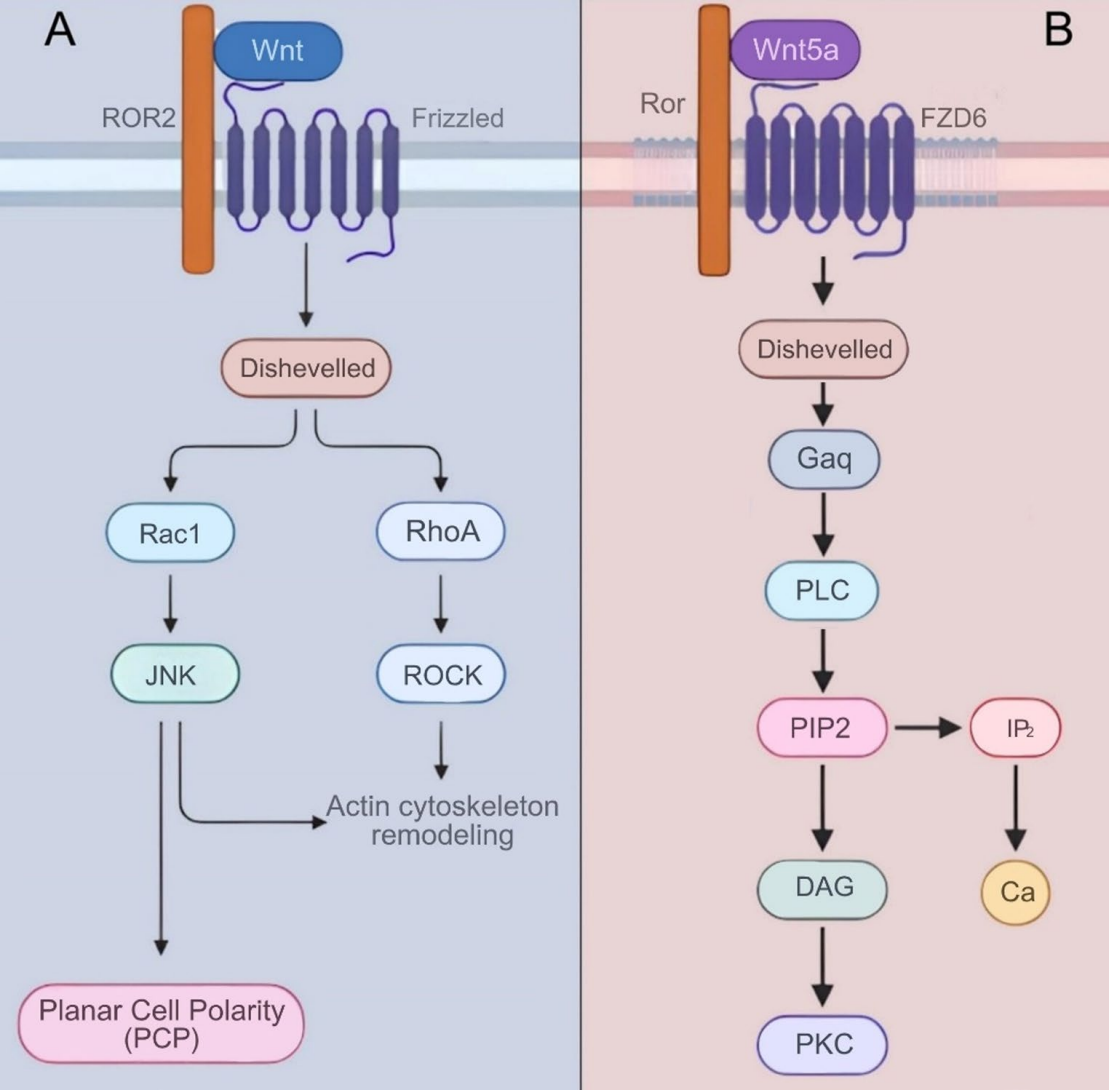
Comparative schematic representation of the Wnt/PCP and Wnt/Ca²⁺ signaling pathways in neural development. *Panel A illustrates the Wnt/planar cell polarity (Wnt/PCP) signaling pathway. Binding of Wnt ligands to Frizzled (FZD) receptors and co-receptors such as ROR2 initiates intracellular signaling through activation of the scaffold protein Dishevelled (DVL). Acting as a central intracellular mediator,* DVL promotes the activation of small Rho GTPases*,* including Rac1 and RhoA. These molecules subsequently trigger downstream kinases such as c-Jun N-terminal kinase (JNK) and Rho-associated protein kinase (ROCK)*,* leading to actin cytoskeleton remodeling. This cascade is essential for the establishment of planar cell polarity (PCP)*,* which underlies key developmental processes including neural tube closure*,* axonal guidance*,* and tissue morphogenesis. Panel B depicts the Wnt/Ca²⁺ signaling pathway. Binding of Wnt ligands*,* particularly Wnt5a*,* to Frizzled receptors (e.g.*,* FZD6) and associated co-receptors activates Dishevelled (DVL)*,* which facilitates G protein–dependent signaling. Activation of the Gαq subunit stimulates phospholipase C (PLC)*,* resulting in the hydrolysis of phosphatidylinositol 4*,*5-bisphosphate (PIP₂) into inositol 1*,*4*,*5-trisphosphate (IP₃) and diacylglycerol (DAG). IP₃ mediates intracellular Ca²⁺ release*,* while DAG together with Ca²⁺ activates protein kinase C (PKC). These signaling events regulate calcium-dependent cellular responses*,* including synaptic activity and neuronal plasticity. This figure was created by the author using BioRender.com*

Functionally, Wnt/PCP signaling aligns neural progenitor cells, promotes convergent extension movements, and directs axon elongation along spatial morphogen gradients [36]. Disruptions in this pathway have been associated with planar cell polarity defects in the neuroepithelium, leading to impaired morphogenesis and contributing to structural anomalies in the developing brain [37]. The mechanistic divergence between Wnt/PCP and Wnt/Ca²⁺ pathways can be visually appreciated in (Fig. 1), which depicts their distinct molecular cascades and downstream targets.

### Wnt/Ca²⁺ pathway: calcium dynamics and neuroregulatory roles

The Wnt/Ca²⁺ pathway is characterized by its capacity to regulate intracellular calcium fluxes in response to non-canonical Wnt ligand stimulation [4]. This pathway is primarily activated by Wnt5a binding to Frizzled receptors and associated co-receptors, which in turn stimulate heterotrimeric G proteins [38]. Subsequent activation of phospholipase C (PLC) leads to the hydrolysis of phosphatidylinositol 4,5-bisphosphate (PIP₂). It generates inositol 1,4,5-trisphosphate (IP₃) and DAG [39]. IP₃ interacts with its receptors on the endoplasmic reticulum (ER), triggering the release of Ca²⁺ ions into the cytoplasm and initiating calcium-dependent signaling cascades [40].

Key downstream effectors of this pathway include PKC, which modulates synaptic vesicle cycling and receptor trafficking; CaMKII, which plays a critical role in memory consolidation and neuronal differentiation; and NFAT, a transcription factor that regulates gene expression associated with synaptic plasticity and neuroinflammation ([41, 42].

Through the concerted actions of these signaling molecules, Wnt/Ca²⁺ signaling contributes to synaptogenesis, neurotransmitter release, and glial cell modulation [43]. It also plays an essential role in maintaining calcium homeostasis in excitable neurons. Dysregulation of this pathway has been implicated in pathological processes such as neuronal hyperexcitability, oxidative stress, and chronic neuroinflammation [44].

**Fig. 2:**
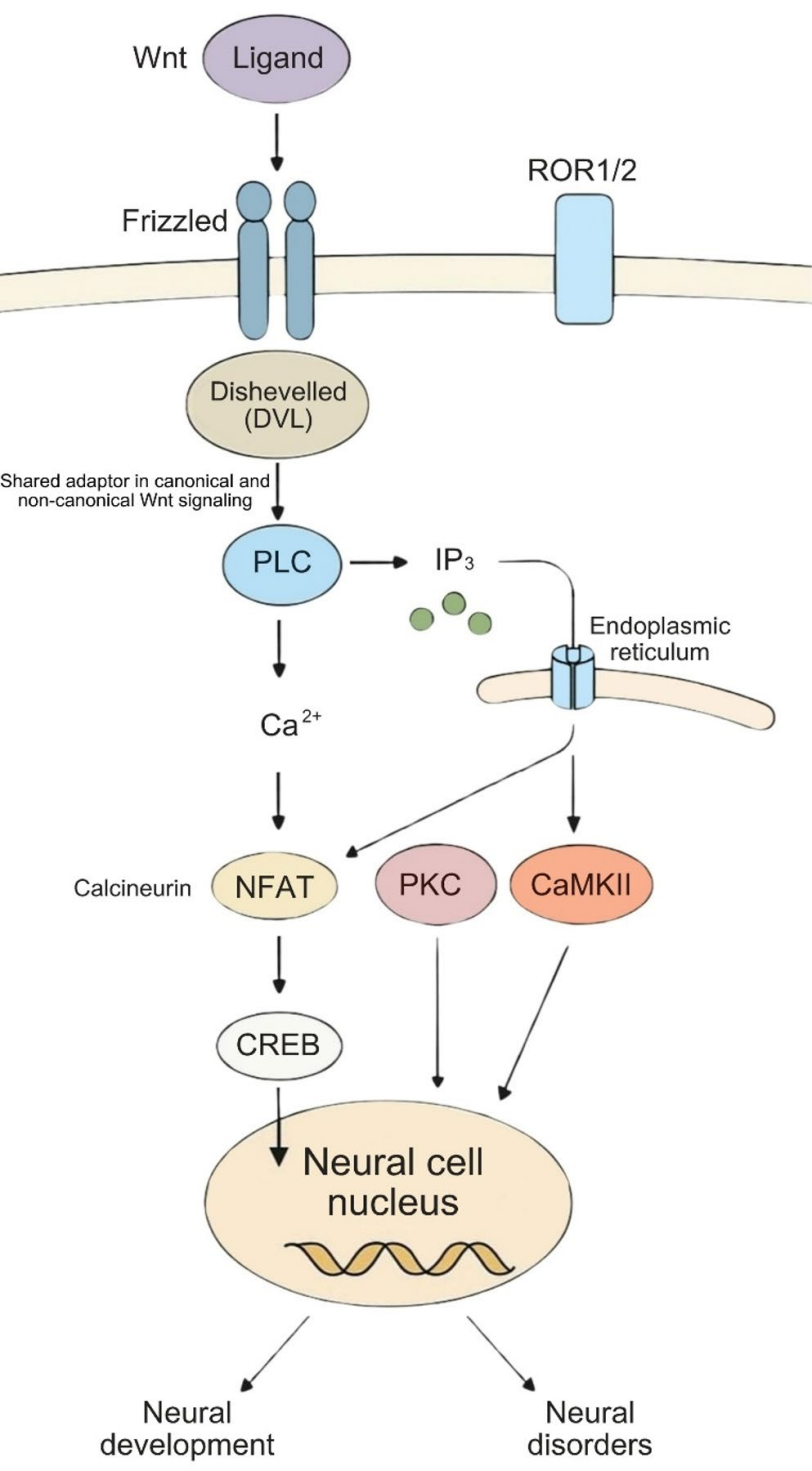
Schematic overview of the non-canonical Wnt/Ca²⁺ signaling pathway and its downstream transcriptional regulation in neural cells. *This schematic depicts the Wnt/Ca²⁺ signaling cascade initiated by the binding of Wnt ligands to Frizzled receptors and co-receptors such as ROR1/2. Upon ligand–receptor interaction,* the intracellular adaptor protein Dishevelled (DVL)*,* which functions as a shared mediator in both canonical and non-canonical Wnt pathways*,* is activated. DVL subsequently promotes phospholipase C (PLC) signaling*,* leading to the generation of inositol 1*,*4*,*5-trisphosphate (IP₃). IP₃ induces the release of Ca²⁺ from the endoplasmic reticulum*,* resulting in elevated intracellular calcium levels. Increased Ca²⁺ activates multiple downstream effectors*,* including calcineurin*,* which regulates nuclear factor of activated T-cells (NFAT)*,* as well as protein kinase C (PKC) and calcium/calmodulin-dependent protein kinase II (CaMKII). These signaling pathways converge on transcriptional regulators such as CREB*,* ultimately modulating gene expression within the neural cell nucleus. Through these mechanisms*,* Wnt/Ca²⁺ signaling contributes to the regulation of neural development and may also be implicated in the pathogenesis of neurological disorders. Abbreviations: Wnt*,* Wingless/Integrated; FZD*,* Frizzled; ROR1/2*,* receptor tyrosine kinase-like orphan receptor 1/2; DVL*,* Dishevelled; PLC*,* phospholipase C; IP₃*,* inositol 1*,*4*,*5-trisphosphate; ER*,* endoplasmic reticulum; Ca²⁺*,* calcium ion; NFAT*,* nuclear factor of activated T-cells; PKC*,* protein kinase C; CaMKII*,* calcium/calmodulin-dependent protein kinase II; CREB*,* cAMP response element-binding protein. This figure was created by the author using BioRender.com*

The involvement of intracellular Ca²⁺ as a second messenger in Wnt signaling leads to the activation of multiple effectors including NFAT, PKC, and CaMKII, which mediate transcriptional changes in neural cells (Fig. 2).

## Comparison: canonical vs. non-canonical pathways in neural contexts

Although both canonical and non-canonical Wnt signaling pathways are initiated by similar ligands and receptor complexes, they exhibit fundamental differences in their downstream signaling cascades, transcriptional outputs, and developmental functions within the nervous system [1, 2, 45]. The canonical Wnt/β-catenin pathway primarily regulates gene transcription through nuclear translocation of β-catenin, thereby influencing neural progenitor proliferation and differentiation [6]. In contrast, non-canonical pathways such as Wnt/PCP and Wnt/Ca²⁺ operate through β-catenin-independent mechanisms that govern cell polarity, cytoskeletal remodeling, calcium homeostasis, and spatial patterning during neurodevelopment [46].

Understanding the mechanistic distinctions as well as the functional interplay (or crosstalk) between these pathways is essential for elucidating how Wnt signaling coordinates complex neural processes such as morphogenesis, axonal guidance, and the etiology of neurodevelopmental disorders.

### Divergence in signal transmission and downstream outcomes

The canonical Wnt pathway is defined by its dependence on β-catenin stabilization and nuclear translocation, where β-catenin forms complexes with T-cell factor/lymphoid enhancer-binding factor (TCF/LEF) transcription factors to initiate the expression of Wnt target genes [47, 48]. In the nervous system, this transcriptional cascade plays a pivotal role in regulating neural stem cell maintenance, proliferation, and early lineage specification [49].

By contrast, non-canonical Wnt pathways-primarily Wnt/PCP and Wnt/Ca²⁺-operate independently of β-catenin and do not directly induce gene transcription [50]. Instead, they activate alternative intracellular signaling mechanisms to modulate cell behavior [51]. The Wnt/PCP pathway primarily governs cytoskeletal architecture by activating small Rho GTPases and JNK, thereby regulating convergent extension, cellular alignment, and planar polarity [26]. The Wnt/Ca²⁺ pathway, on the other hand, controls calcium dynamics through IP₃-mediated Ca²⁺ release and subsequent activation of effectors such as PKC, CaMKII, and NFAT. These signaling modules regulate neuronal excitability, synaptogenesis, and glial communication [6].

This mechanistic divergence allows non-canonical Wnt pathways to mediate spatially localized, temporally rapid, and transcription-independent cellular responses [52]. Such properties are particularly critical during dynamic developmental processes including neuroepithelial morphogenesis, neuronal migration, and axon pathfinding contexts in which precise subcellular coordination is required [53].

Despite their shared use of upstream Wnt ligands and Frizzled family receptors, canonical and non-canonical Wnt signaling differ markedly in their downstream cascades and biological outputs (Table 1). The canonical β-catenin-dependent pathway results in transcriptional activation, while non-canonical branches achieve their effects through cytoskeletal or calcium-regulated signaling routes, enabling more immediate and context-specific responses during neural development.

**Table 1:** Comparative Features of Canonical and Non-canonical Wnt Signaling in Neural Contexts

Feature	Canonical Wnt	Wnt/PCP	Wnt/Ca²⁺
Core Receptors	FZD ⁺ LRP5/6	FZD ⁺ ROR2/PTK7 context-dependent)	FZD ± co-receptors (e.g., ROR2)
Downstream Key Effector	β-catenin	RhoA, JNK (DVL-mediated)	PLC, Ca²⁺ (DVL-mediated)
Output Mechanism	Gene transcription	Cytoskeletal remodeling	Ca²⁺-dependent signaling
Key Biological Roles	Proliferation, fate	Migration, polarity	Synaptic plasticity
Related Disorders	Colon cancer, leukemia	Neural tube defects (e.g., spina bifida), ASD	Alzheimer’s, epilepsy

### Crosstalk and co-regulation in neurodevelopment

Despite their distinct molecular mechanisms, canonical and non-canonical Wnt signaling pathways frequently interact, exhibiting either antagonistic or synergistic relationships depending on cellular context, receptor availability, and developmental stage [54]. This crosstalk plays a pivotal role in fine-tuning neural development by coordinating the timing and specificity of transcriptional versus cytoskeletal responses [46].

One notable mechanism of interaction involves the Frizzled (Fzd) family of receptors, which can initiate either canonical or non-canonical signaling depending on the expression of co-receptors such as Low Density Lipoprotein Receptor Related Protein 5/6 (LRP5/6) for canonical β-catenin signaling, or ROR2 and PTK7 for non-canonical branches [55, 56]. Wnt5a, a prototypical non-canonical ligand, exemplifies functional duality by suppressing β-catenin activity in certain contexts while simultaneously activating Ca²⁺-dependent pathways in others [57]. DVL, a cytoplasmic scaffold protein common to both arms of Wnt signaling, serves as a central regulatory node that integrates upstream inputs and directs downstream signaling depending on its post-translational modifications and interaction partners [58].

Temporally, canonical Wnt signaling is predominantly active during early stages of neurodevelopment, where it supports neural progenitor proliferation, maintenance of stemness, and lineage commitment [59]. As development progresses, non-canonical signaling becomes more prominent, orchestrating spatial organization, cytoskeletal remodeling, neuronal migration, and synaptic circuit formation [60].

Importantly, disruption of this balance has been implicated in several neurodevelopmental disorders [13]. In conditions such as schizophrenia, spina bifida, and neural tube defects, aberrant crosstalk between the canonical and non-canonical Wnt axes has been observed [61]. These findings underscore the necessity of tightly regulated signaling dynamics to ensure precise spatial and temporal patterning during central nervous system development.

## Outcome: functional and pathological consequences of dysregulated signaling

The spatial and temporal precision of Wnt/PCP and Wnt/Ca²⁺ signaling is indispensable for orchestrating neural development, circuit formation, and synaptic plasticity [6]. Dysregulation of these non-canonical pathways leads to a spectrum of molecular, cellular, and anatomical abnormalities that underlie various neurodevelopmental and neurodegenerative conditions [37].

At the cellular level, these signaling defects may manifest as actin cytoskeleton disorganization, calcium imbalance, and loss of neuronal polarity. At the systems level, they contribute to functional disconnectivity and structural anomalies within neural circuits [62]. Understanding the pathological consequences of dysregulated Wnt signaling not only clarifies the etiological underpinnings of these disorders but also highlights key molecular targets for potential therapeutic intervention [63].

### Developmental impairments: from morphogenesis to miswiring

One of the most essential roles of Wnt/PCP signaling lies in the establishment of PCP, a prerequisite for neural tube closure during early embryonic development [26]. Genetic mutations affecting PCP core components including VANGL1/2, DVL, and Frizzled receptors such as Frizzled Class Receptor 3 (FZD3) and Frizzled Class Receptor 6 (FZD6) have been strongly associated with NTDs such as spina bifida, craniorachischisis, and anencephaly [64, 65].

The specificity of Wnt/PCP signaling in neural development is not solely determined by ligand–receptor interactions but also depends on the spatial organization of core PCP components within distinct cellular contexts. Members of the CELSR (Cadherin EGF LAG seven-pass G-type receptor) family play essential roles in planar cell polarity, neural tube closure, and axon guidance [66–68].

Experimental evidence suggests that CELSR proteins contribute to the organization of PCP complexes in a context-dependent manner, which may influence how signaling outputs are interpreted across different neural cell populations. This context dependency provides a plausible explanation for the broader phenotypic spectrum observed in FZD3-related defects compared to more restricted phenotypes in other PCP mutants. Importantly, cell type–dependent organization of PCP components provides a mechanistic basis for how signaling outputs are differentially regulated in proliferating neural progenitors versus postmitotic neurons.

Consistent with these mechanistic insights, original human and experimental studies further support the involvement of Wnt/PCP components in neural development. Targeted sequencing studies in human NTD cohorts have identified rare damaging variants in CELSR family genes and related PCP components as significant contributors to disease susceptibility. Experimental studies in vertebrate models have likewise demonstrated that disruption of Frizzled-mediated PCP signaling impairs neural tube closure and planar polarity [69, 70]. These structural malformations reflect underlying failures in cellular alignment, tissue elongation, and convergent extension movements regulated by PCP signaling [71].

Beyond morphogenesis, disruptions in Wnt/PCP signaling also compromise neuronal migration and axon guidance, resulting in cortical mislamination and impaired neural wiring [72]. These architectural deficits are increasingly implicated in the etiology of complex neurodevelopmental disorders, including ASD, schizophrenia, and intellectual disability, which are characterized by altered synaptic connectivity and information processing [73].

In parallel, dysregulated Wnt/Ca²⁺ signaling has been shown to interfere with critical processes such as synaptogenesis, dendritic arborization, and activity-dependent plasticity [74]. Perturbations in intracellular Ca²⁺ homeostasis and impaired activation of downstream effectors including PKC and CaMKII lead to deficits in long-term potentiation (LTP), a cellular correlate of learning and memory [75].

Collectively, these findings underscore the pivotal role of non-canonical Wnt signaling in shaping both structural and functional aspects of neurodevelopment [76].

An additional aspect of non-canonical Wnt signaling in neural development is its role in the generation and planar polarization of ependymal motile cilia. Planar cell polarity (PCP) signaling has been shown to regulate the coordinated orientation of cilia, which is essential for directional cerebrospinal fluid flow along the ventricular system [77].

Genetic studies further demonstrate that disruption of core PCP components, particularly Celsr2 and Celsr3, impairs ependymal ciliogenesis and leads to severe defects such as hydrocephalus, highlighting the importance of PCP signaling in maintaining ciliary structure and function [78]. In addition, PCP genes have been shown to exert distinct roles across different neural cell populations, contributing both to progenitor organization and to the positioning and function of differentiated ciliated cells [79].

Together, these findings indicate that non-canonical Wnt/PCP signaling plays a critical role in establishing ciliary polarity and function, and that its disruption can compromise cerebrospinal fluid dynamics and ultimately affect brain homeostasis.

### Neurological disorders associated with Wnt/PCP and Wnt/Ca²⁺ dysregulation

Beyond their developmental roles, dysregulated non-canonical Wnt pathways have been increasingly implicated in the pathophysiology of progressive neurological disorders [37]. In Alzheimer’s disease, hyperactivation of the Wnt/Ca²⁺ signaling axis leads to aberrant intracellular Ca²⁺ accumulation, which in turn activates calcineurin and its downstream transcription factor NFAT. This cascade contributes to synaptic dysfunction, chronic neuroinflammation, and neuronal apoptosis hallmark features of Alzheimer’s pathology [80]. Notably, elevated levels of Wnt5a, a key ligand of the Wnt/Ca²⁺ pathway, have been detected in postmortem brain tissues of Alzheimer’s patients, further supporting its pathogenic role in disease progression [6].

Similarly, in Parkinson’s disease, disrupted Wnt/Ca²⁺ signaling has been shown to impair mitochondrial function, rendering dopaminergic neurons more susceptible to oxidative stress and degeneration. Concurrently, altered PCP signaling within adult neurogenic niches may hinder neurogenesis and regenerative capacity, thereby exacerbating neurodegeneration [81].

The role of Wnt5a-mediated non-canonical signaling in Parkinson’s disease appears to be context-dependent. Experimental studies in dopaminergic neuron models have demonstrated that Wnt signaling can modulate neuronal survival and neuroinflammatory responses in Parkinson’s disease models [82–84]. Under certain conditions, Wnt5a-related signaling pathways have been associated with increased neuronal vulnerability and oxidative stress, whereas in alternative cellular contexts they have been shown to support neuronal survival and adaptive stress responses [84, 85].

These apparently conflicting findings may reflect differences in experimental models, disease stages, and cellular environments, including the balance between canonical and non-canonical Wnt signaling pathways. Collectively, these data indicate that Wnt5a signaling exerts context-specific effects on dopaminergic neuron survival in Parkinson’s disease. However, the role of Wnt5a-mediated signaling in Parkinson’s disease remains complex and context-dependent. While several studies suggest that activation of non-canonical Wnt/Ca²⁺ signaling contributes to dopaminergic neuron vulnerability through calcium dysregulation and oxidative stress, other reports indicate that Wnt5a may exert neuroprotective effects under specific experimental conditions. These protective effects have been linked to modulation of synaptic function, inflammatory responses, and cellular stress adaptation.

These apparently contradictory findings may reflect differences in experimental models, disease stages, and cellular context, including the balance between canonical and non-canonical Wnt signaling pathways. Therefore, the impact of Wnt5a on dopaminergic neuron survival cannot be considered unidirectional, and further studies are required to clarify its context-specific functions in Parkinson’s disease.

Across these disorders, several mechanistic signatures converge, including the loss of cellular polarity and tissue organization, calcium overload-induced excitotoxicity, and impaired synaptic connectivity and plasticity [86]. These molecular aberrations compromise neural circuit integrity and highlight the continued relevance of Wnt/PCP and Wnt/Ca²⁺ signaling beyond embryonic development. Collectively, these findings underscore their dual role as regulators of both developmental processes and lifelong neural homeostasis [87].

## Clinical and translational perspectives

The expanding understanding of Wnt/PCP and Wnt/Ca²⁺ signaling in neural development and pathology opens promising translational pathways for both therapeutic innovation and disease modeling [88]. As these non-canonical routes regulate essential processes such as neural tube closure, synaptic formation, and intracellular calcium homeostasis, their targeted manipulation could yield interventions for both neurodevelopmental and neurodegenerative disorders [89].

### Therapeutic targets within non-canonical Wnt pathways

Therapeutic modulation of non-canonical Wnt signaling is an emerging frontier in neuropharmacology, with several promising strategies currently under investigation [90]. One potential target is DVL, a scaffold protein that mediates signal propagation in both the Wnt/PCP and Wnt/Ca²⁺ pathways [91]. Inhibitors of DVL are being explored to suppress hyperactive signaling, particularly in neurodegenerative contexts such as Alzheimer’s disease, where aberrant Wnt activity exacerbates pathological cascades [4].

Another strategy involves the development of Frizzled receptor modulators, including monoclonal antibodies and synthetic peptides, that can interfere with Wnt ligand binding or selectively bias downstream signaling toward either the canonical or non-canonical branch. Such approaches offer the potential to finely tune Wnt signaling based on disease-specific demands, avoiding blanket suppression that could disrupt essential developmental or homeostatic functions [92].

At the level of intracellular effectors, pharmacological modulation of NFAT and CaMKII has gained traction as a means to restore calcium homeostasis and synaptic integrity in cognitive disorders. These targets are particularly relevant in conditions where Wnt/Ca²⁺ signaling is hyperactive and linked to excitotoxicity or synaptic disassembly [93].

Potential clinical applications of these strategies include suppression of neuroinflammation in Alzheimer’s disease, restoration of neuronal polarity in ASD, and modulation of synaptic plasticity in epilepsy and schizophrenia [94]. Nevertheless, achieving pathway specificity remains a significant challenge due to the pleiotropic and highly context-dependent nature of Wnt signaling, necessitating the development of precision-targeted interventions with minimal off-target effects [95].

### Experimental models and organoid systems

To elucidate the mechanisms of non-canonical Wnt signaling and facilitate the screening of potential therapeutic agents, a variety of advanced model systems have been developed [24]. Human brain organoids derived from iPSCs offer a powerful platform for modeling human-specific aspects of neurodevelopment [96].

Clustered Regularly Interspaced Short Palindromic Repeats/CRISPR-associated protein 9 (CRISPR/Cas9-mediated) gene editing has further expanded the ability to dissect the functional roles of individual signaling molecules [97]. Knock-in or knock-out models targeting effectors such as Prickle or CaMKII can be applied both in vivo using zebrafish and murine models and in vitro, within neural progenitor cell lines [98]. Together, these experimental platforms offer an integrative framework for investigating non-canonical Wnt signaling in both developmental and pathological contexts, as summarized in (Table 2).

**Table 2:** Translational Tools and Models for Studying Wnt/PCP and Wnt/Ca²⁺ Signaling

Model/Tool	Application Area	Pathway Targeted	Notes
Brain organoids	Developmental modeling	Both	Mimics spatial polarity and 3D neural organization
CRISPR/Cas9 KO mice	Genetic validation	PCP or Ca²⁺	Enables component-specific functional dissection
Live Ca²⁺ imaging (GCaMP)	Functional pathway monitoring	Wnt/Ca²⁺	Real-time visualization of intracellular calcium flux
DVL inhibitor (e.g., NSC668036)	Pharmacological modulation	Both	Selectively targets shared scaffolding protein
Zebrafish embryo system	In vivo morphogenetic analysis	Wnt/PCP	Used to study convergent extension and polarity defects

## Conclusion

Non-canonical Wnt signaling, mediated through the Wnt/PCP and Wnt/Ca²⁺ pathways, plays a central role in regulating neural development, cellular organization, and synaptic function. These pathways contribute to critical processes such as neural tube closure, neuronal migration, and synaptic plasticity, and their dysregulation is implicated in a wide range of neurological disorders, including neurodevelopmental and neurodegenerative diseases.

This review provides an integrated perspective on the molecular architecture and functional roles of Wnt/PCP and Wnt/Ca²⁺ signaling in the nervous system. By comparatively examining these pathways and their crosstalk with canonical Wnt signaling, we highlight their context-dependent functions and their contribution to both physiological and pathological processes. In addition, the synthesis of recent findings emphasizes the importance of non-canonical Wnt signaling as a dynamic regulatory system rather than a linear signaling cascade.

Despite these advances, several critical challenges continue to limit the direct translation of non-canonical Wnt signaling into clinical applications. The context-dependent nature of Wnt signaling, extensive pathway crosstalk, and incomplete understanding of cell type–specific responses complicate therapeutic targeting. Further research is required to clarify these mechanisms and to develop more precise and context-specific interventions.

## Data Availability

No datasets were generated or analysed during the current study.

## Abbreviations

Wnt: Wingless/Integrated
PCP: Planar Cell Polarity
Ca²⁺: Calcium Ion
NFAT: Nuclear Factor of Activated T-cells
PKC: Protein Kinase C
CaMKII: Calcium/Calmodulin-Dependent Kinase II
CRISPR: Clustered Regularly Interspaced Short Palindromic Repeats
iPSCs: Induced Pluripotent Stem Cells
NTDs: Neural Tube Defects
DVL: Dishevelled
ROR2: Receptor Tyrosine Kinase-Like Orphan Receptor 2
JNK: c-Jun N-terminal Kinase
ASD: Autism Spectrum Disorder
PK: Prickle
FZD3: Frizzled Class Receptor 3
FZD6: Frizzled Class Receptor 6
DAG: Diacylglycerol
GTPases: Guanosine Triphosphatases
LRP5: Low Density Lipoprotein Receptor Related Protein 5
LTP: Long-Term Potentiation
TCF: T-cell Factor
PTK7: Protein Tyrosine Kinase 7
VANGL: Van Gogh-like
VANGL1: Van Gogh-like Protein 1
RYK: Receptor-like Tyrosine Kinase

## Declarations

### Generative AI Use

The authors declare that ChatGPT (OpenAI, GPT-5.2) was used exclusively for language polishing and minor grammatical corrections during manuscript preparation.
